# A Comprehensive Study about the Role of Crosslink Density on the Tribological Behavior of DLC Coated Rubber

**DOI:** 10.3390/ma13235460

**Published:** 2020-11-30

**Authors:** Suleyman Bayrak, Dominik Paulkowski, Klaus Werner Stöckelhuber, Benjamin Staar, Bernd Mayer

**Affiliations:** 1 Fraunhofer Institute for Manufacturing Technology and Advanced Materials IFAM, 28359 Bremen, Germany; dominik.paulkowski@ifam.fraunhofer.de (D.P.); bernd.mayer@ifam.fraunhofer.de (B.M.); 2 Leibniz-Institut für Polymerforschung Dresden IPF, 01069 Dresden, Germany; stoeckelhuber@ipfdd.de; 3 Bremer Institut für Produktion und Logistik BIBA, 28359 Bremen, Germany; sta@biba.uni-bremen.de; 4 Faculty of Production Engineering, University of Bremen, 28359 Bremen, Germany

**Keywords:** rubber materials, rubber friction, plasma technology, DLC coating, polymer characterization, elastomer surface, tribology

## Abstract

The friction and wear behavior of coated rubber components is strongly dependent on the substrate properties. This work deals with the impact of the crosslink density, i.e., the hardness of the rubber substrate on the tribological performance of uncoated and coated rubber. The hardness of nitrile butadiene rubber (NBR) is varied altering the sulfur content. Both the uncoated and coated rubber samples are characterized in terms of surface and mechanical properties. Tribological tests comprise the examination of the macroscopic contact area and the temperature in the contact zone. It was found that the functional layer enhances the wear resistance significantly. Apparently, the wear and friction behavior of the coated rubber correlates with the hardness and the bulk properties of the substrate material.

## 1. Introduction

Rubber seals became a key element in many technical applications, e.g., in the automotive and aviation industry [1]. Oil-based lubricants minimize the wear of the seals and ensure their functionality. The increasing demand for lubrication-free protection of those rubber components accelerates the development of environment-friendly alternatives [2]. Recent developments leverage diamond-like carbon (DLC, amorphous carbon *a*-C:H) films as an attractive solution regarding environmental protection and energy saving [3,4,5,6]. Unlike fluid lubricants, DLC coatings can be used even under extreme vacuum conditions without losing their excellent tribological behavior [1].

Polymeric materials, notably rubber materials, are sensitive to temperature. Therefore, plasma-enhanced chemical vapor deposition (PECVD) is widely employed to deposit the hard amorphous carbon on the rubber surface [7]. The adhesion strength and flexibility of DLC coatings on rubber has been investigated under different conditions [7,8,9]. The works approve the good compatibility of the amorphous carbon on the rubber substrates and a constantly low coefficient of friction under dynamic test conditions.

The first publication about DLC coating on polymeric materials was made by Nakahigashi et al. Radio frequency-plasma assisted chemical vapor deposition (RF-PECVD) was applied to deposit DLC coating (1 µm) on various polymeric materials. For NBR material, the coefficient of friction (CoF) could be reduced from *µ* = 1.6 to 0.7 (bearing steel, Ø 8 mm, 0.5 N) [5].

Martinez-Martinez investigated the coating segmentation of hydrogenated acrylonitrile butadiene rubber (HNBR) during the PECVD process. They found that the segmentation can be controlled by the bias voltage during the deposition process. The segmentation originates from the substrate temperature during the deposition process and is beneficial for the flexibility of the layer [10].

Bui et al. presented a very good adhesion strength of DLC coatings deposited on HNBR. Even after stretching the sample up to 50% and unloading, no delamination or spallation could be found. The thin layer of 1 µm exhibited a very low CoF of 0.2 compared to 1.5 (100Cr6 ball, Ø 6 mm, 3 N) [8].

Pei et al. reduced the CoF of NBR from *µ* = 1 to 0.25 by employing an expanding thermal plasma-based deposition. Their investigation showed that a high arc current leads to high coating hardness, smaller patch sizes and better adhesion [11].

Masami et al. deposited Si-DLC coating on fluoro rubber at low temperature (30 K) by means of a bipolar pulsed plasma-based ion implantation (PBII). The CoF could be reduced from 1.9 down to 0.2 (steel ball, Ø 3.13 mm, 0.49 N) [12].

By changing the surface properties, the rubber friction itself becomes a worth-considering impact. Kummer proposed the unified theory of rubber friction, which has been developed successively over the course of the past decades [13,14,15,16,17,18,19]. According to the theory of rubber friction, the frictional force can be split into three main components: The adhesive, hysteresis, and cohesion component. The adhesive friction force arises due to attachments and detachment of molecular bondings between the surface of the rubber sample and the counterpart [20]. Asperities and rough surfaces lead to deformation and energy dissipation. This internal damping effect accounts for the hysteresis-based component of the rubber friction [15]. The friction force, caused by cohesive failure of the material, is called as the cohesive fraction. It is assumed to be negligible compared to the previous mentioned main contributing factors [13,14,15,16,17,18,19,20,21].

The adhesion component of smooth rubber samples can be reduced by functionalizing the surface with a thin DLC film [22]. However, there have been a few studies examining the influence of different rubber properties on the tribological behavior of DLC coatings [4,23]. Paulkowski et al. investigated three rubber types (ethylene propylene diene monomer rubber (EPDM), fluoroelastomer (FKM) and acrylonitrile butadiene rubber (NBR)) with the same hardness (75 ShA). The CoF was measured in an oscillating tribological set up (100Cr6, Ø 10 mm, 4.7 N) and could reportedly be reduced for all three rubber types by depositing a DLC layer of 1 µm. The authors manageed to find differences in the wear rate depending on the substrate material. FKM seems to have a lower wear rate than EPDM and NBR [4].

Thirumulai et al. present in their studies three elastomeric substrates, FKM, NBR and thermoplastic urethane (TPU). Regarding the substrate properties, the authors discuss the tribological properties of the substrate materials under the shed of the static contact depth of the substrate materials and conclude that the rigidity of the substrates must be higher for lower contact depth. The authors postulate a high coefficient of friction for the more rigid substrate [23].

It should be emphasized, that the mentioned papers compare different rubber types, which may have an impact on the frictional behavior. Furthermore, the authors do not consider the mechanical properties of the rubber substrates under dynamic conditions. This aspect should be relevant, if we discuss dynamic tribological tests. Therefore, we take dynamic mechanical analysis (DMA) of the following different rubber materials of the same types into account.

In this work, we vary the sulfur content of nitrile butadiene rubber and investigate the relations between the rubber hardness and the functional layer. During sulfur vulcanization, the macromolecules of the rubber are cross-linked in a rather complex chemical reaction by polysulfide bridges in a three-dimensional network. In addition to these chemical network nodes, the physical entanglement of the macromolecules must also be taken into consideration. A variation in the sulfur content therefore changes the crosslinking density of the rubber, which affects the static and dynamic properties of the material, which are reflected, for example, in the storage and loss modulus, stiffness, and hardness of the elastomer material [24]. We demonstrate the importance of the bulk properties for the tribological performance and the wear behavior of the DLC layer.

## 2. Experimental Procedure

### 2.1. Materials, Pretreatment, and Deposition

As basic polymer nitrile butadiene rubber (NBR) was chosen. A NBR grade with an acrylonitrile content of 34.7% (Perbunan 3446F) was kindly supplied by Arlanxeo (Dormagen, Germany). Sulfur, zinc oxide, and stearic acid were acquired from Acros Organics (Schwerte, Germany). The vulcanizing accelerator di(benzothiazol-2-yl) disulfide (MBTS) was delivered by Lanxess (Köln, Germany). Carbon black N330 was kindly provided by Orion Engineered Carbons, Germany.

Compounding was carried out in a laboratory internal mixer Thermo Haake Rheocord PolyLab 600p (Thermo Electron, Karlsruhe, Germany), containing the NBR rubber, zinc oxide, stearic acid, and carbon black. The starting temperature was 100 °C and the total mixing time was 7 min. The raw mix was homogenized on a two-roll mixing mill (Polymix-110L, Servitec GmbH, Wustermark, Germany) at 50 °C with a constant friction ratio of 1:1.2. In this second mixing step, the vulcanization chemicals were added on the mixing mill.

The crosslink density of the rubber and in consequence its different hardness was controlled by varying the sulfur content (1.5, 3, 6, and 9 phr, part per hundred rubber). The amount of reinforcing carbon black (40 phr) was kept constant and plasticizers were not incorporated for all samples. Additional ingredients are summed up in Table 1. The stored rubber compounds were rheometrically analyzed in a rubber process analyzer (Scarabaeus SIS-V50, Scarabaeus GmbH, Wetzlar, Germany) to determine their optimal vulcanization time T90. Vulcanization of these rubber samples was conducted then at 160 °C into sheets of 2 mm thickness in a heated compression molding press (Fontijne TP1000, Delft, Netherlands) with a force of 600 kN for optimum vulcanization time T90 plus 1 min per mm thickness of the sample. A polished steel plate was inserted in the vulcanization mold in order to obtain ideally smooth rubber surfaces (arithmetical mean height *Sa* = 9.8 nm ± 0.34). Both, the polished plate and the mold, were coated by a release layer.

A dissolved tenside detergent was used in an ultrasonic cleaning procedure in order to remove contaminations from the rubber surface [25]. The sonicated specimens were rinsed with demineralized water and, subsequently, stored 24 h at room temperature.

Hard amorphous carbon was deposited by means of PECVD. The capacitively coupled plasma (CCP) is driven by radio-frequency (13.56 MHz) with an asymmetric set up. The deposition process started with a short surface pretreatment activation using Argon (supplied by Linde, 99.999% purity) as precursor. A thin layer, formed by tetramethylsilane (TMS, (CH_3_)_4_Si, supplied by Acros Organics, 99.9% purity) as precursor, improves the adhesion and reduces the internal stresses [26]. The functional layer was deposited with toluene (C_6_H_5_CH_3_, supplied by neofroxx, purity 99.9%) and TMS for the DLC coating (thickness 1 µm). Table 2 shows a condensed overview of the deposition parameters and the ratio of the components during the deposition process.

The rubber plates were coated along with silicon and glass substrates, respectively. The latter were used to measure the coating hardness and the surface free energy.

### 2.2. Substrate Properties

Dynamic-mechanical measurement were performed at a frequency of 10 Hz in the tensile mode using a Netzsch-Gabo Eplexor 2000 N. The relevant data were recorded in the temperature range between −60 to 140 °C with steps of 2 °C. Above glass transition temperature *T_g_*, the oscillating mode was set between 0.5% and 1.5%. The specimen size was 10 × 35 mm^2^.

Uniaxial stress strain behavior was studied according to DIN 53504 using a universal testing machine Zwick Z010. For each sample, two reflection points (distance between points 15 mm) mark the strain length for the optical strain measurement. Three specimens were stretched at a speed of 200 mm/min.

DIN ISO 2781 was applied for the determination of the density of the rubber materials. The weight of three rubber specimens (size 10 × 10 mm^2^) per material was measured in air and water and calculated according to Archimedes’ principle (Metter Toledo XS603s precision scale).

The Shore A hardness of the substrate materials was measured according to DIN ISO 7619.

### 2.3. Coating and Surface Analysis

The hardness of the deposited coating was investigated on silicon substrates. A Universal Material Tester (UMT1, Bruker, USA) equipped with a Berkovich-Indenter was used. For each sample, ten measurement points were taken into consideration and a maximum force of 0.5 mN was chosen. The distance between each point was 50 µm.

Like the coating hardness, the thickness of the coating was measured on silicon substrate. The ellipsometer WVA SE32 by LOT-Quantum was used. A wavelength spectrum from 500 to 1000 nm with a range of 25 nm was chosen and the angle of incidence was set from 65 to 75°.

The surface free energy was measured with a measuring device for contact angles, named Krüss G2 (Krüss GmbH, Hamburg, Germany). Six liquids (water, diiodomethane, ethylene glycol, glycerol, decane, and benzyl alcohol) were applied in order to define the polar and dispersive part of the surface free energy according to [27].

The confirmation and classification for the amorphous structure of the DLC coating was proved by Raman analysis. A micro-Raman spectroscope from Renishaw (System 1000) with a wavelength of 514 nm (2.4 eV) was used, provided with an argon ion laser. The laser spot has a diameter of 10 µm. The Raman spectrum was evaluated according to Ferrari et al. [28,29].

In order to determine the hydrogen content, coating powder was stored 24 h at 100 °C. Subsequently, the powder was combusted in an oxygen stream at 1050 °C. The combustion water was analyzed by means of infrared spectroscopy.

The topography of the uncoated rubber samples was characterized by an atomic force microscope (AFM, EasyScan 2, Nanosurf, Liestal, Switzerland). Operating in the tapping mode, the AFM is particularly suitable for the measurements of the uncoated rubber plates, since we do expect ideally smooth surfaces. Three areas with an image size of 90 × 90 µm^2^ were taken into account. 

The coated rubber surfaces were measured optically with a confocale microscope (Sensofar, PLu neox, Terrassa, Spain). The relatively rough surface of the coated surface can fully be detected with the scanning size of 180 × 250 µm^2^.

SEM images and EDS analyses were conducted with a Phenom XL (Thermo Fisher Scientific, Waltham, MA, USA).

### 2.4. Tribology

**Figure 1:**
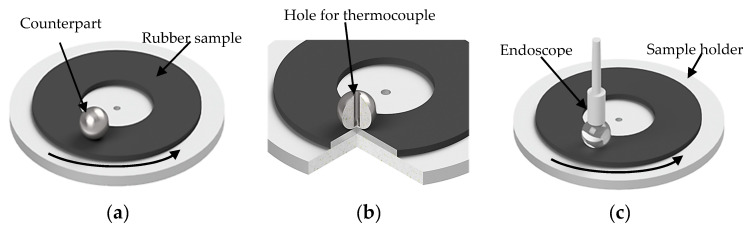
Scheme of tribological set-up: (**a**) configuration to measure CoF; (**b**) sphere with hole for temperature measurement; (**c**) configuration to measure the nominal contact area with glass ball.

The ball-on-disc configuration (Figure 1a) was selected for tribological test. The rubber samples were mounted on the steel sample holder and placed into the Universal Material Tester UMT3. The lower drive operates in rotating mode against a steel ball. Table 3 contains the friction parameters. The tests were performed in a hermetic system with a relative humidity of *rH* = 31.07 ± 1.01%. The CoF of each sample was measured once.

The tribological tests include the temperature measurements in the contact zone. A thermocouple (type K) was implemented in the steel ball at a distance of *d* = 0.5 mm to the surface (Figure 1b). Further sliding tests were performed with a glass ball to investigate the macroscopic contact area (Figure 1c).

## 3. Results and Discussion

### 3.1. Substrate Properties

**Figure 2:**
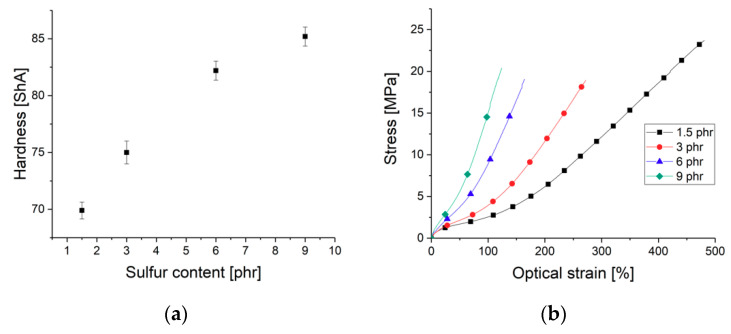
Nitrile butadiene rubber: (**a**) Shore A hardness in dependence on the sulfur content according to DIN ISO 7619; (**b**) stress–strain curves for different sulfur contents.

The hardness values at room temperature are summed up in Figure 2a. The increasing sulfur content causes a higher crosslink density constraining the chain motion and segment re-configurations [24,30]. Likewise, the modulus is governed by the amount of sulfur during the vulcanization process. As shown in Figure 2b, the static stress–strain curve gets steeper for the highly crosslinked samples indicating a higher modulus. A clear tendency regarding the tensile strength cannot be deduced with the given data.

**Figure 3:**
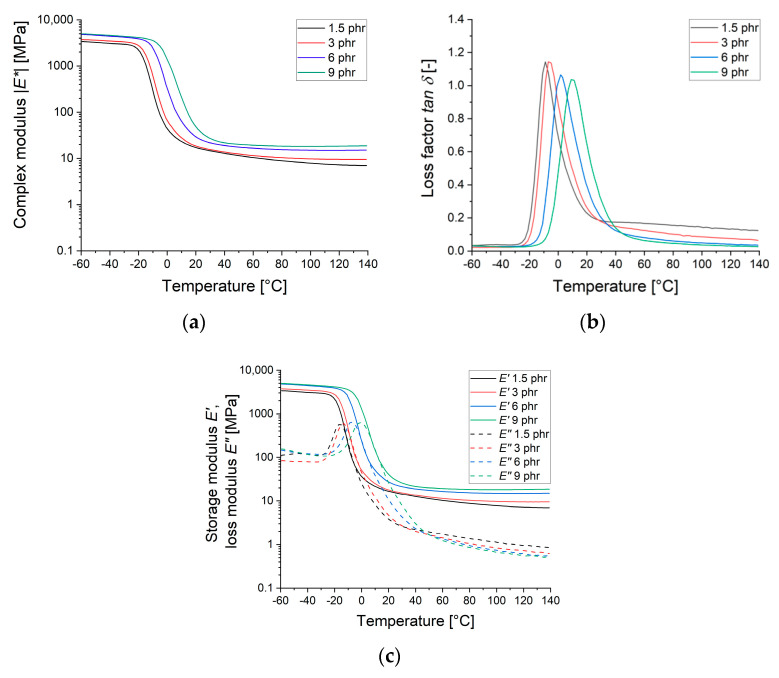
Temperature-dependent dynamic–mechanical curves for different sulfur contents: (**a**) Complex modulus $\left|E^{*}\right|;\;$(**b**) loss factor tanδ; (**c**) storage modulus E’ and loss modulus $E^{\prime \prime }$.

Further mechanical–dynamical analysis provide insight into the rubber properties under dynamic strain conditions at various temperatures. The rubber samples with higher sulfur content exhibit a higher complex modulus $\left|E^{*}\right|\;$(Figure 3a) on the rubbery plateau. In the latter region, the modulus declines slightly stronger for samples with lower sulfur content. Moving from very low temperatures to higher temperatures the glass transition temperature *T_g_* follows a clear tendency, as illustrated in Figure 3b. The higher the sulfur content, the higher the glass transition temperature *T_g_* (−8.9, −6.9, 1.8, and 9 °C). The area above *T_g_* starts at lower temperatures and extends across a higher temperature range for lower sulfur contents.

Altogether, the increasing amount of sulfur reduces the loss factor and the loss modulus in the rubbery region (Figure 3c).

An approximation between the storage modulus *E’* and the crosslink density $\vartheta_{e}$ is given [24] by Equation (1):

(1) $$E^{\prime }=3\vartheta_{e}RT$$

where *E’* is the storage modulus at the rubbery plateau, $\vartheta_{e}$ the crosslink density and *R* the gas constant with 8.314 J/(K·mol). The temperature *T* was chosen as 298 K.

**Figure 4:**
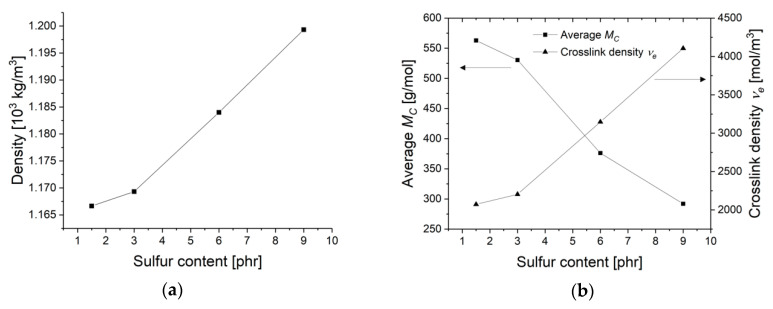
(**a**) density ρ; (**b**) average molecular weight between crosslinks *Mc* and crosslink density $\vartheta_{e}$ at various sulfur content.

The denity of the rubber materials increases with higher sulfur content, as it can be seen in Figure 4a. Taking the density *ρ* (kg/m^3^) into account, the average molecular weight between two crosslinks *M_c_* (g/mol) can be written [24]:

(2) $$E’=\frac{3\rho RT}{M_{c}}$$

The average molecular weight between two crosslinks *M_c_* decreases from 562 to 292 g/mol whereas the crosslink density $\vartheta_{e}$ increases from 2072 to 4100 mol/m^3^ for the sample with the lowest (1.5 phr) to the highest sulfur content (9 phr) (Figure 4b).

### 3.2. Coating Properties

**Figure 5:**
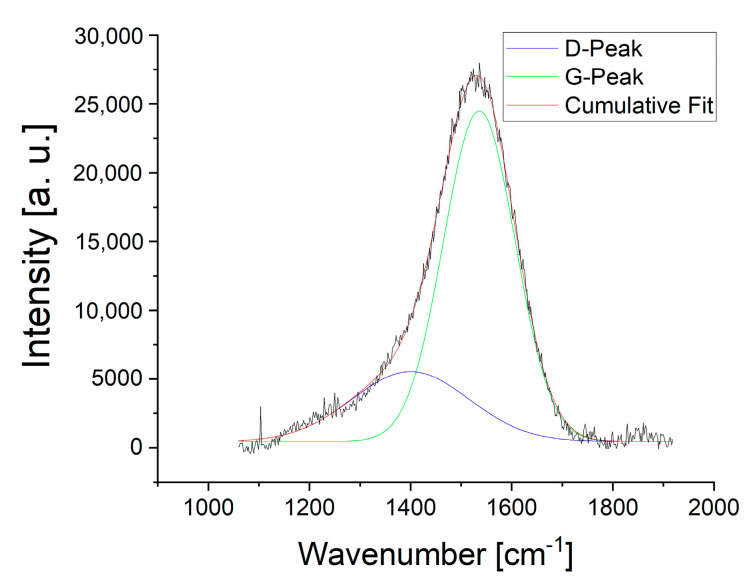
Raman spectrum of the deposited coating with an excitation wavelength of 514 nm.

Figure 5 plots the Raman spectrum of the deposited coating. The Raman spectrum exhibits the D- and G-Peak, which are characteristic for amorphous structure. The G-Peak is located 1527 cm^−1^. The ratio of *ID/IG* is 0.24. Comparing these results with the literature, we can estimate the sp^3^ to have fraction of ~0.4 [28,29]. Komori et al. deposited toluene-based DLC coatings with various ratio of TMS by PECVD [31]. They studied the carbon bond structure by Raman spectroscopy with an excitation wavelength of 532 nm. It was found that the G-peak position shifts to lower values from 1527 to 1506 cm^−1^ if TMS is added to the deposition process. The intensity ratio of D- and G-Peak shifts to lower values from 0.36 for the pure toluene to 0.32 for the mixed toluene/TMS process [31]. Near edge X-ray absorption fine structure (NEXAFS) indicate a small intensity ratio compared to tetrahedral amorphous carbon (ta-C). Thus, the sp^3^ fraction is expected to be much lower than ta-C coatings [31]. Further structural investigations on toluene-based DLC coatings deposited by PECVD [32,33], plasma-based ion implantation (PBII) [34,35,36] and ion vapor deposition [37] can be found in the literature, respectively.

The properties of the coating were determined previously [4,25]. Its Young’s modulus is *E* = 77.5 ± 9.5 GPa (9 ± 1.1 GPa hardness) and surface free energy is 33.41 mN/m, whereby polar part is 7.37 mN/m.

The hydrogen content is 37.8 at % and was measured by combusting coating powder and analyzing the combustion water (see chap. 2.3). Komori et al. characterized hydrogenated DLC by Rutherford backscattering spectroscopy (RBS). The hydrogen content remained with 36 at % unchanged for toluene-based and toluene/TMS-based coatings [31]. Further literature on the hydrogen content of toluene-based DLC is pointed out for PECVD [32], PBII process [24,34,35,38], and ion vapor deposition [39]. It is well known that the hydrogen content plays a role in the tribological behavior of DLC coating [6]. Since we apply only one coating type in the subsequent tribological tests, we expect the bonded hydrogen content to stay constant.

### 3.3. Roughness and Surface Topography

**Figure 6:**
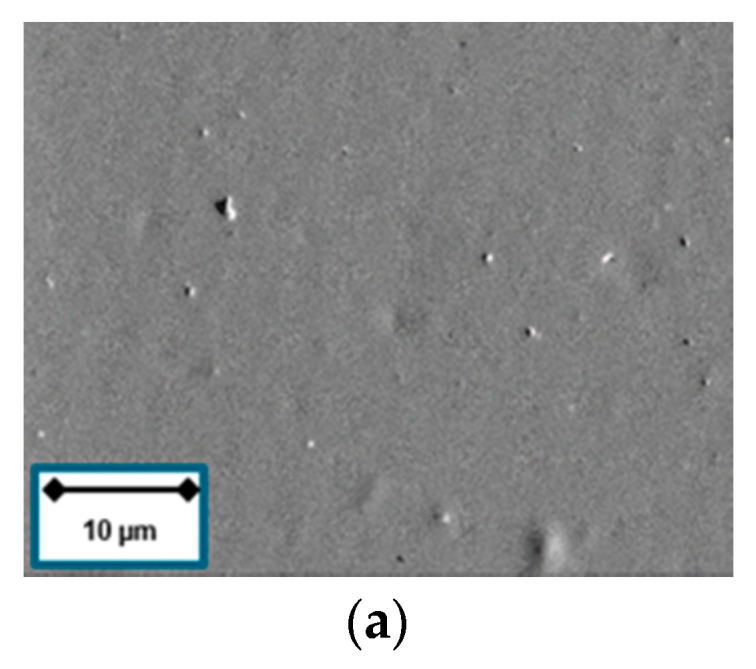

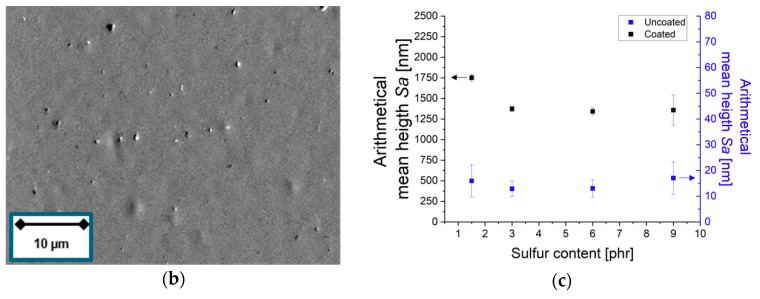
(**a**) 1.5 phr surface condition before Ar pretreatment; (**b**) 1.5 phr surface condition after Ar pretreatment; (**c**) arithmetical mean height *Sa* of uncoated and coated NBR samples at different sulfur content.

Polymeric materials may be damaged if exposed to UV radiation and ion irradiation. Figure 6a,b show exemplarily the surface condition of the softest material vulcanized with 1.5 phr sulfur. No damage could be detected after the short argon (Ar) pretreatment.

The uncoated rubber surfaces exhibit a constantly low arithmetical mean height *Sa* between *Sa* = 13 nm (3 phr) and *Sa* = 17 nm (9 phr), as it can be derived from Figure 6c. There is no clear correlation between sulfur content and the surface roughness for the uncoated sample. By contrast, the roughness of the coated samples might be influenced by the substrate properties. The rubber sample compounded with 1.5 phr sulfur has the highest roughness (*Sa* = 1752 nm) after deposition compared to *Sa* = 1342 nm for 6 phr and 1358 nm for 9 phr, respectively.

**Figure 7:**
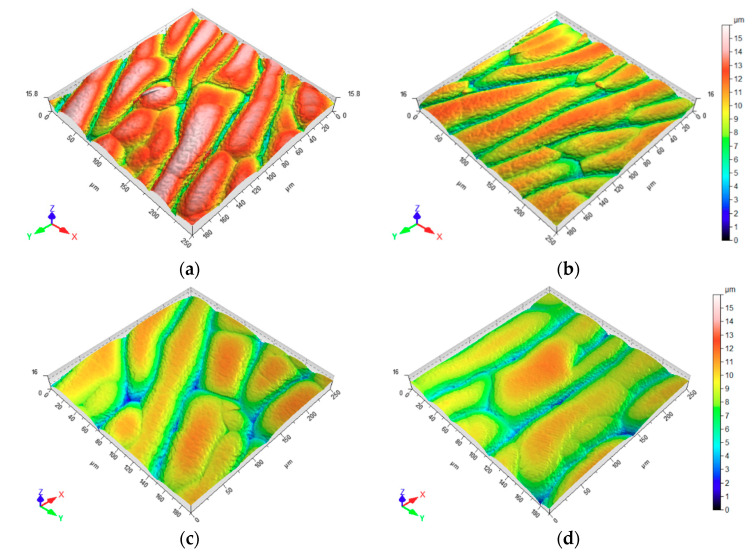
Surface plot of coated nitrile butadiene rubber (NBR) after deposition: (**a**) 1.5 phr; (**b**) 3 phr; (**c**) 6 phr; and (**d**) 9 phr sulfur content.

The coating surface topography of the coated rubber is governed by the internal stress and the temperature the sample is exposed to during the deposition process [40]. Assuming the soft material is less restrictive to thermal expansion, the material elongation causes more cracks in the DLC film ending up in small patch sizes after contraction. It can be deduced that harder materials, i.e., samples with higher sulfur content, show larger patch sizes, as it can be seen in Figure 7a–d.

### 3.4. Friction Measurement, Temperature in Contact Zone, and Contact Area

**Figure 8:**
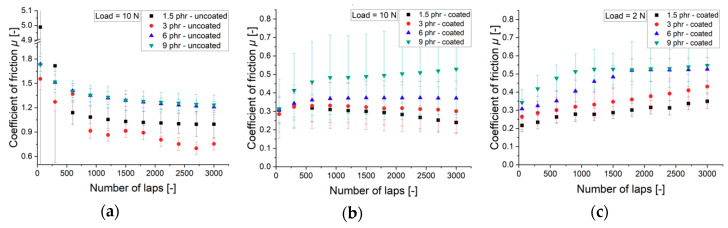
Coefficient of friction *µ*: (**a**) uncoated rubber samples with 10 N; (**b**) coated samples with a load of 10 N; (**c**) coated samples with a load of 2 N.

Figure 8a depicts the frictional behavior of the NBR without being functionalized by the DLC coating. In this short-term friction test, the running-in period is reached after 1000 laps. Hereafter, no transition in CoF can be detected between the samples. The soft material, compounded with 1.5 phr sulfur content, stands out with a remarkably high CoF during the first 300 laps. The reason for this is the stick-slip effect. Apart from the softest sample, the CoF shows more or less a clear tendency depending on the substrate hardness. The CoF increases with the sulfur content.

The CoF curves for the coated NBR samples are provided in Figure 8b. The running-in period is clearly shorter. A steady state is reached after 600 laps. In addition, the friction appears to be lower at the beginning for all coated samples. This fact is not consistent to Figure 8a, where the CoF starts at higher values and declines during the course of the test. For 1.5 and 3 phr samples, the CoF even falls slightly, whereas it rises in case of the 9 phr substrate.

Obviously, the CoF of the coated NBR has the same correlation as the uncoated samples. The CoF increases with the sulfur content. Following order is reached after 3000 laps for the DLC coated substrates: (*µ_1.5phr,DLC_* = 0.23; *µ_3phr,DLC_* = 0.3, *µ_6phr,DLC_* = 0.36, and *µ_9phr,DLC_* = 0.53). That implies, that the friction is rather governed by the bulk property of the substrate material. The same correlation between substrate and the CoF could be found for a lower load of 2 N, as it can be derived from Figure 8c. The CoF increases with the substrate hardness. The CoF exhibits high error bars. The high velocity of 1200 mm/s is responsible for this systematic error.

**Figure 9:**
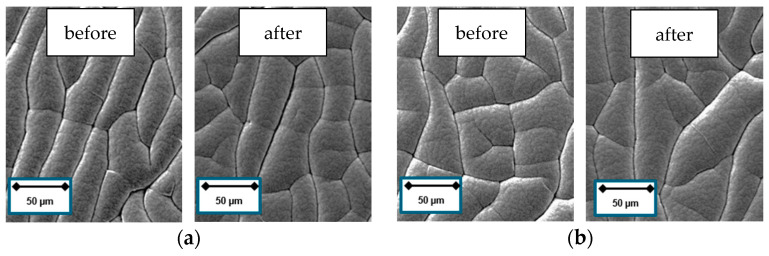
Surface condition: (**a**) Coated 1.5 phr substrate before and after initial approach with 10 N; (**b**) coated 9 phr substrate before and after initial approach with 10 N.

Figure 9a,b indicate the surface condition of the coated rubber samples after the initial approach with a load of 10 N. No changes could be detected neither for the softest nor for the hardest substrate material.

**Figure 10:**
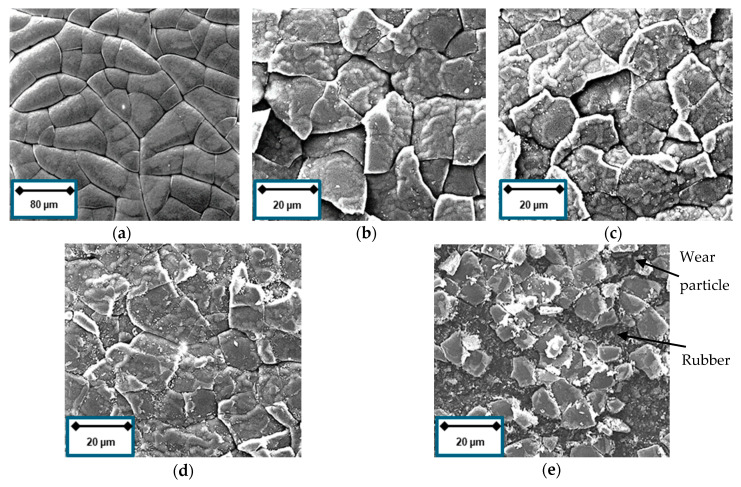
SEM images of coated NBR: (**a**) 1.5 phr before tribotest; (**b**) 1.5 phr; (**c**) 3 phr; (**d**) 6 phr; and (**e**) 9 phr after 3000 laps.

Figure 10a shows the surface of the unaffected DLC coated NBR. After the tribotest and at higher magnification, crack formation can be observed for all types of coated samples (Figure 10b–e). The crack formation does not seem to interfere with the functional task of the coating, namely the friction reduction. Furthermore, the amount of wear particle increases with the hardness of the rubber material indicating a substrate-dependent abrasion of the DLC coating. The DLC film, coated on soft material, benefits from the high damping factor and is less prone to abrasive wear. The substrate compounded with the highest amount of sulfur exhibits severe defects after the tribotest.

**Figure 11:**
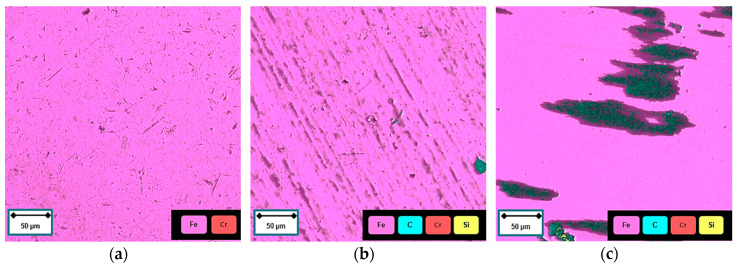
EDX mapping of the 100Cr6 counterpart (**a**) before tribotest; (**b**) after tribotest on 1.5 phr coated specimen; and (**c**) after tribotest on 9 phr coated specimen.

Figure 11 depicts the EDX mapping of the counterpart before the tribotest Figure 11a and after tribotest for the 600 V coated softest (1.5 phr, Figure 11b) and hardest material (9 phr, Figure 11c). In contrast to the virgin counterpart, both the used counterparts reveal the presence of carbon and silicon on the surface, which is assumed to originate from the coating. Large embedded rubber remains can be detected on the counterpart employed during the tribotest on the hardest substrate.

**Figure 12:**
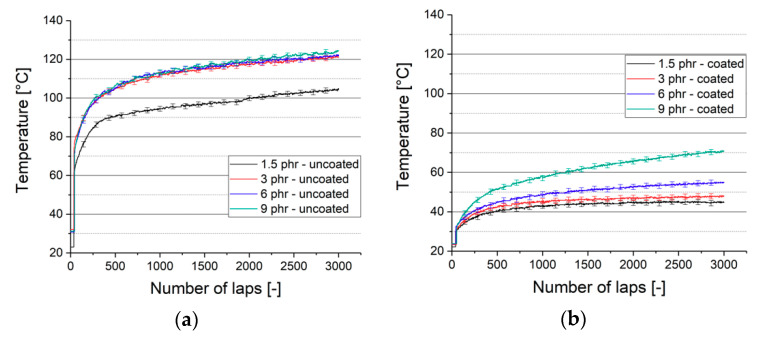
Temperature in contact zone during friction test: (**a**) uncoated rubber samples; (**b**) coated rubber samples.

Higher friction leads to an increase of energy losses in form of heat and temperature rises in the contact zone. Indeed, uncoated samples with a higher CoF exhibit also higher temperatures in the contact zone, as shown in Figure 12a. While the softest material reaches *T_1.5phr_* = 102 °C after 3000 laps, the differences between the higher crosslinked samples are relatively smaller (*T_3phr_* = 121, *T_6phr_* = 122 and *T_9phr_* = 124 °C).

The friction reduction by the DLC coating leads to lower temperatures in the contact zone (Figure 12b). We observe a steady temperature increase for the hardest substrate (9 phr). This finding correlates with the linearly increasing CoF (Figure 8b). The curves flattens with decreasing substrate hardness due to low friction and wear formation.

The values do not represent the actual temperature in the contact zone, but rather an approximation. The real temperature is expected to be higher [41]. Yet, the fact that differences are detectable, reinforces the measuring technique as an appropriate method.

**Figure 13:**
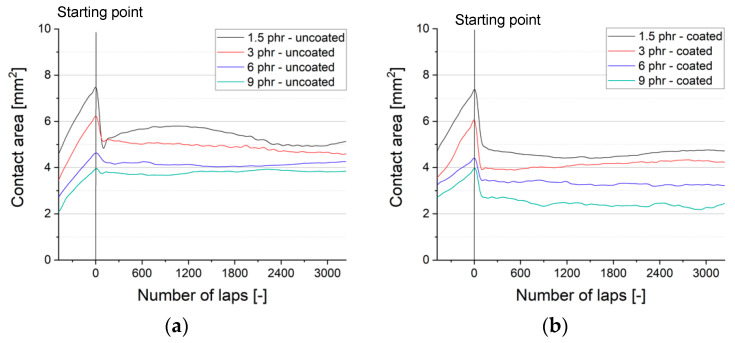
Effect of substrate on the nominal contact area between rubber sample and counterpart: (**a**) uncoated rubber samples; (**b**) coated rubber samples.

The metrology to monitor the contact area between the sample and the counterpart under dynamic conditions was introduced in reference 42. As described in Table 3 and Figure 1, a glassy ball was inserted for the following tests. We have shown that the referenced approach is a reliable measuring technique with an average standard deviation of 3% [42]. The results for the contact area of uncoated samples are shown in Figure 13a. It can be verified, that the soft material exhibits constantly a larger contact area (*A_1.5phr_* = 5.4 mm^2^) than the harder materials during the friction test. The value falls to *A_3phr_* = 5.1, *A_6phr_* = 4.2 and ends up with *A_9phr_* = 3.8 mm^2^ for the hardest rubber sample. 300 laps was chosen as point of reference, where we expect a steady state with a comparatively low influence of wear and debris formation.

**Figure 14:**
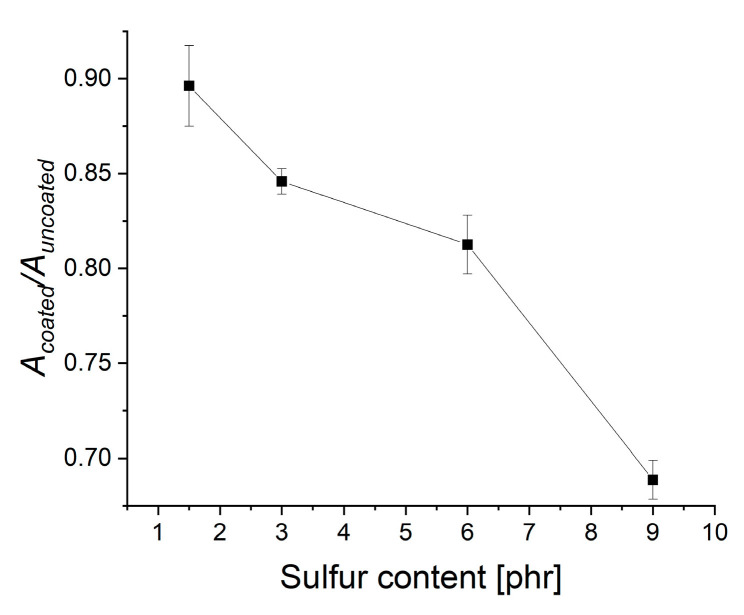
Ratio of contact area for coated and uncoated samples at various sulfur content.

The declining tendency can also be found for the coated rubber samples (Figure 13b); an indication, that the nominal contact area is still governed by the substrate. The soft substrate accounts for the largest contact area *A_1.5phr,DLC_* = 4.7 mm^2^, less crosslinked materials *A_3phr,DLC_* = 3.9, *A_6phr,DLC_* = 3.4 and *A_9phr,DLC_* = 2.7 mm^2^. A closer study of the results reveals that the gap between the individual samples becomes higher. Particularly for the hard substrate material, the ratio of the nominal contact area declines significantly (see Figure 14).

## 4. Summary and Conclusions

A major gap has been found in the existing literature regarding DLC coated rubber: There have been no studies about the effect of the substrate properties on the friction and wear behavior of DLC coated rubber. For the first time, we present DLC coating deposited on the same rubber type with different mechanical properties. In order to control the entire process chain, the functionalization of the rubber surface with DLC coating comprises the vulcanization of the rubber plates. We varied the mechanical properties of NBR by altering the sulfur content. The increasing sulfur content leads to a higher crosslink density and, therefore, higher hardness. The CoF, the temperature in the contact zone as well as the nominal contact area of coated samples show the same correlation as the uncoated samples. The CoF increases with the substrate hardness, both for coated and uncoated rubber samples. The CoF of all four rubber samples could be reduced significantly. For the softest NBR the reduction is up to 76% (0.99 to 0.24) and 42% for the hardest NBR (1.23 to 0.53). The temperature in the contact zone rises for higher crosslinked substrates, whereas the contact area exhibits higher values for softer substrates. Less damping substrate materials are more prone to abrasive wear formation and exhibit more wear. 

It should be noted, that this work does not explain the wear mechanisms of the DLC coating.

Unlike in previously mentioned studies in the existing literature, we used for the first time ideally smooth rubber surfaces. That allows us to conclude, that the different topography and the roughness emerge as a result of the different substrate hardnesses. Further investigations on the quantitative contribution of the different coating topography to the tribological behavior are pending.

## Figures and Tables

**Table 1:** Ingredients of the examined materials.

Ingredient	Amount [phr ^1^]
NBR rubber (Perbunan 3446F)	100
Carbon black N330	40
Zinc oxide ZnO	5
Stearic acid	1.5
MBTS	1.5
Sulfur	variable: 1.5, 3, 6, 9

^1^ Parts per hundred rubber.

**Table 2:** Deposition parameters at 4 × 10^−3^ mbar basic pressure; 1 sccm = 1.667 × 10^−9^ m^3^/s.

Deposition Parameters	Pretreatment			Interlayer			Functional Layer		
Precursor	Gas flow	Bias	Time	Gas flow	Bias	Time	Gas flow	Bias	Time
	[sccm]	[V]	[s]	[sccm]	[V]	[s]	[sccm]	[V]	[s]
Argon	30	400	120	-	-	-	-	-	-
Tetramethylsilane (TMS)	-	-	-	20	600	120	20	600	480
Toluene	-	-	-	-	-	-	80	600	480

**Table 3:** Tribological parameters.

Load	Velocity	Temperature	Ball diameter	Material
[N]	[mm/s]	[°C]	[mm]	100Cr6 /
10	1200	30	10	Borosilicate
